# Seasonality of Glacial Snow and Ice Microbial Communities

**DOI:** 10.3389/fmicb.2022.876848

**Published:** 2022-05-16

**Authors:** Matthias Winkel, Christopher B. Trivedi, Rey Mourot, James A. Bradley, Andrea Vieth-Hillebrand, Liane G. Benning

**Affiliations:** ^1^GFZ German Research Centre for Geosciences, Helmholtz Centre for Geosciences, Potsdam, Germany; ^2^School of Geography, Queen Mary University of London, London, United Kingdom; ^3^Department of Earth Sciences, Freie Universität Berlin, Berlin, Germany

**Keywords:** glacier ice algae, snow algae, seasonality, microbial dynamics, cryosphere

## Abstract

Blooms of microalgae on glaciers and ice sheets are amplifying surface ice melting rates, which are already affected by climate change. Most studies on glacial microorganisms (including snow and glacier ice algae) have so far focused on the spring and summer melt season, leading to a temporal bias, and a knowledge gap in our understanding of the variations in microbial diversity, productivity, and physiology on glacier surfaces year-round. Here, we investigated the microbial communities from Icelandic glacier surface snow and bare ice habitats, with sampling spanning two consecutive years and carried out in both winter and two summer seasons. We evaluated the seasonal differences in microbial community composition using Illumina sequencing of the 16S rRNA, 18S rRNA, and ITS marker genes and correlating them with geochemical signals in the snow and ice. During summer, *Chloromonas*, *Chlainomonas*, *Raphidonema*, and *Hydrurus* dominated surface snow algal communities, while *Ancylonema* and *Mesotaenium* dominated the surface bare ice habitats. In winter, algae could not be detected, and the community composition was dominated by bacteria and fungi. The dominant bacterial taxa found in both winter and summer samples were *Bacteriodetes*, *Actinobacteria*, *Alphaproteobacteria*, and *Gammaproteobacteria*. The winter bacterial communities showed high similarities to airborne and fresh snow bacteria reported in other studies. This points toward the importance of dry and wet deposition as a wintertime source of microorganisms to the glacier surface. Winter samples were also richer in nutrients than summer samples, except for dissolved organic carbon—which was highest in summer snow and ice samples with blooming microalgae, suggesting that nutrients are accumulated during winter but primarily used by the microbial communities in the summer. Overall, our study shows that glacial snow and ice microbial communities are highly variable on a seasonal basis.

## Introduction

Glaciers and ice sheets cover roughly 10% of Earth’s land surface and store approximately 69% of Earth’s freshwater (Meredith et al., 2019), thus serving as a critical natural resource. Moreover, glaciers and ice sheets are important components of Earth’s climate system—responding to global temperature changes, and are a major driver of sea level change (Meier et al., 2007). There has been increased surface melting of glaciers and ice sheets in recent decades (Nghiem et al., 2012; Lenaerts et al., 2013) resulting from both land surface air temperature increases as well as reductions in glacier surface albedo (Box et al., 2012; Ji et al., 2014; Cook et al., 2020; Tedstone et al., 2020)—a change to which the biodiversity of glacier and glacier adjacent habitats is sensitive (Anesio et al., 2017; Cauvy-Fraunié and Dangles, 2019; Stibal et al., 2020). Pigmented algae inhabiting glacier and ice sheet surfaces are part of the light absorbing particulates present on snow and ice surfaces. Such particulates have been shown to decrease surface albedo and increase surface melt rates of both snow and bare ice surfaces (Benning et al., 2014; Dumont et al., 2014; Lutz et al., 2016; Cook et al., 2020; Williams et al., 2020). Among these particulates, pigmented snow and glacier ice algal blooms play a major role in changing the albedo during the late spring to late summer melt season. In the dark zone on the western margin of the Greenland Ice Sheet, glacier ice algae contribute between 13% and 26% of the total annual surface melt (Cook et al., 2020) by lowering the albedo. As the main phototrophic primary producers on glacier surfaces, algae are also important for carbon and nutrient cycling (Anesio et al., 2017) as they produce dissolved organic carbon (DOC; Hood et al., 2009, 2015; Singer et al., 2012) that acts as a substrate for heterotrophic organisms (Stibal et al., 2012) and fertilizes downstream ecosystems when exported in meltwater (Lawson et al., 2014; Musilova et al., 2017; Irvine-Fynn et al., 2021).

Algae are found in numerous different habitats on glaciers, including cryoconite holes (Hamilton et al., 2013; Lutz et al., 2019), snow (Lutz et al., 2015a; Havig and Hamilton, 2019), bare ice (Yallop et al., 2012; Lutz et al., 2016, 2018; Williamson et al., 2018), biofilms (Lutz et al., 2017), and glacial streams (Smith et al., 2017). Such pigmented algae are often visible in distinct red or deep purple blooms on the snow and glacial ice surfaces (Lutz et al., 2016, 2018; Cook et al., 2020; Stibal et al., 2020; Williamson et al., 2020). The most commonly reported green and red snow algae are those of the phyla *Chlorophyta* and *Ochrophyta*, and the genera *Chloromonas*, *Chlamydomonas*, and *Raphidonema* (Lutz et al., 2015a), while *Hydrurus* has been reported as the dominant genus in yellow snow (Remias et al., 2013). In contrast, algal communities on bare ice surfaces are typically dominated by the phylum *Streptophyta* (*Zygnematophyceae*) consisting primarily of *Mesotaenium* (recently reclassified as *Ancylonema alaskana*; Procházková et al., 2021) and *Ancylonema nordenskioeldii* (Remias et al., 2009; Yallop et al., 2012; Lutz et al., 2018). Bacteria, archaea, fungi, and viruses are also found in glacial surface habitats (Larose et al., 2010; Maccario et al., 2014; Bellas et al., 2015; Lutz et al., 2015b; Perini et al., 2019). Most of the bacteria and fungi inhabiting glacier surfaces are heterotrophs that can feed on algal exudates (Lutz et al., 2015b), and they drive important biogeochemical processes including the fixation of atmospheric nitrogen (Telling et al., 2011; Larose et al., 2013).

Studies of microbial processes in supraglacial ecosystem have been limited to the spring and summer melt seasons, and an evaluation of variation in summer vs. winter dynamics is lacking. Challenges associated with sampling glacial and high-latitude environments during winter conditions include inaccessibility of sampling sites, adverse weather and light conditions, and low rates of biological activity (Carpenter et al., 2000). Furthermore, deep snow tends to cover glacier surface during winter, making the ice surface less accessible for sampling. Bacterial communities in snowpacks have been well characterized, even in early spring prior to melt (Larose et al., 2010; Maccario et al., 2014), however knowledge of the seasonal dynamics of bacterial, archaeal, fungal, viral, or algal communities is poor. Microbial communities have been analyzed seasonally in other cryospheric environments, including the active layers of permafrost (Schostag et al., 2015), thermokarst lake water columns (Vigneron et al., 2019), and alpine soils (Lazzaro et al., 2015), yet these systems differ significantly from glacial ice and snow and therefore offer little to no comparison of biological, chemical and physical processes.

Here, we assessed the microbial community composition and nutrient variations of glacier surface ice and snow habitats on various Icelandic glaciers, with sampling carried out during two consecutive summers and one winter time period. We analyzed the structure of glacial microbial communities using bacterial and eukaryotic marker genes and measured physico-chemical parameters including the concentrations of DOC, major ions, pH, and conductivity. For eukaryotic communities, we used two marker genes (18S rRNA and ITS) as previous studies (Lutz et al., 2018; Engstrom et al., 2020) showed these to give a more complete overview of eukaryotic diversity. Our data revealed distinct differences between summer and winter supraglacial microbial communities and we discuss their links to variable geochemical signatures of the surface snow and ice environments.

## Materials and Methods

### Field Sites, Sampling, and Sample Handling

**Figure 1:**
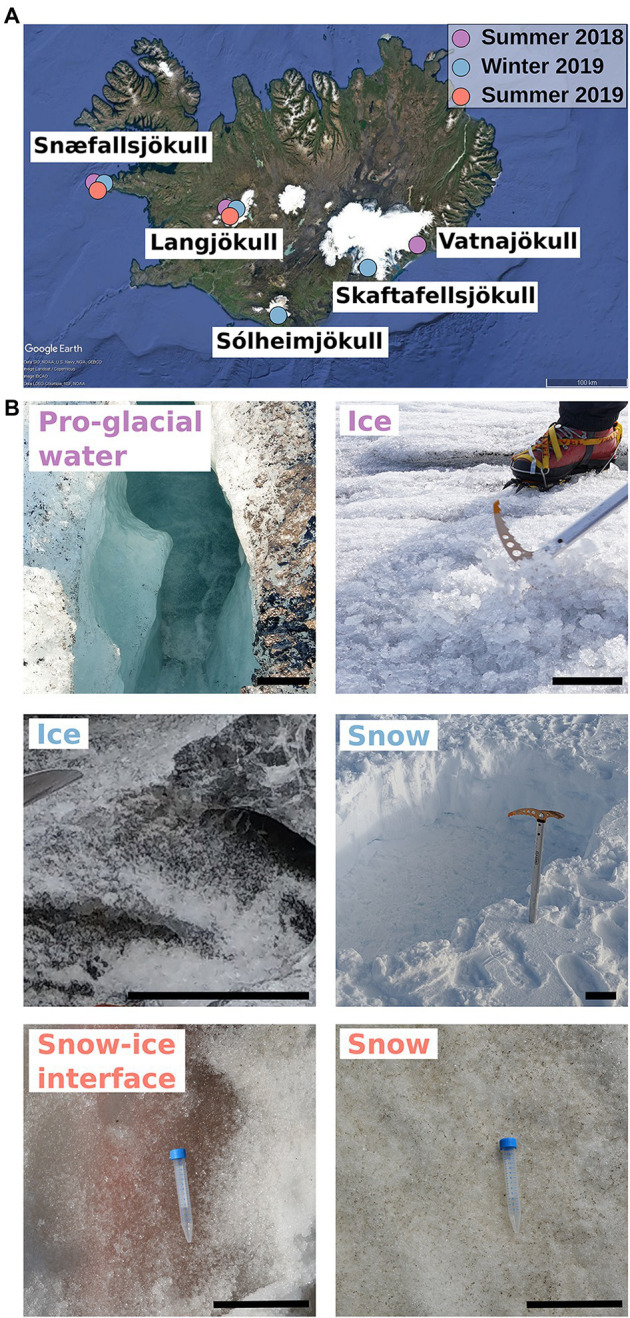
Sampling collection sites and habitats in Iceland. **(A)** Map of Iceland showing the geographical locations and time of season of the glaciers sampled for this study. **(B)** Images of representative habitats collected in the different seasons (colors; ice summer 2018 © J. Perez, snow winter 2018 © C. Trivedi, snow–ice interface, snow summer 2019 © J. Bradley, pro-glacial water summer 2018, ice winter 2019 © M. Winkel). The scale bar on all photographs represents 12 cm.

Snow and ice samples were collected from four glaciers and one ice cap in Iceland (Figure 1) during the summers in 2018 (September) and 2019 (August), and in the winter 2019 (February; Table 1). Not all locations were sampled during each time point and snow/ice sample pairs could not always be collected at each site. In particular, during the 2019 winter sampling at Snæfellsjökull and Langjökull, only fresh snow could be collected. In summer, additional samples representing snow–ice interfaces were collected at Langjökull (2018) and Snæfellsjökull (2019), and pro-glacial waters were collected only at Langjökull (2018). The top few cm of 10 snow, seven ice, two snow–ice interface habitats, and one pro-glacial water sample were collected into sterile whirl pack bags or into 50 ml sterile Falcon® tubes using pre-sterilized plastic or metal trowels. The samples were used for marker gene sequencing, solution and particulate analysis, pH, and conductivity. For DOC analysis, separate samples were collected with sterile metal trowels into acid-washed (~10% HCl, 24 h) and ashed (550°C, 6 h) glass jars.

**Table 1:** Overview of samples, locations, habitats, coordinates, and field measurements.

Season	Sample ID	Location	Habitat	Date (DD/MM/YY)	Latitude (dd.dd dd°)	Longitude (dd.dd dd°)	Elevation (masl)	pH	Conductivity (μs cm^−1^)	Filtered volume for DNA (ml)	Air temperature (°C)	Rel. humidity (%)
September 2018 (summer)	IS18-1	Snæfellsjökull	Ice	06/09/18	64.8121	−23.7394	783	5.88	2.94	50^a^	n.a.	n.a
	IS18-2	Snæfellsjökull	Snow (d)	06/09/18	64.8132	−23,7,380	773	5.95	5.93	50a.	n.a.	n.a.
	IS18-3	Langjökull	Ice	07/09/18	64.6360	−20.5595	853	5.84	2.46	50^a^	n.a.	n.a.
	IS18-4	Langjökull	Snow–ice Interface	07/09/18	64.6360	−20.5595	853	5.94	2.61	50^a^	n.a.	n.a.
	IS18-5	Langjökull	Snow (d)	07/09/18	64.6360	−20.5595	853	5.76	2.50	50^a^	n.a.	n.a.
	IS18-6	Langjökull	Pro-glacial water	07/09/18	64.6435	−20.5690	880	6.02	2.65	150^b^	n.a.	n.a.
	IS18-7	Vatnajökull	Snow (d)	09/09/18	64.2595	−15.8614	786	5.52	5.31	50^a^	n.a.	n.a.
	IS18-8	Vatnajökull	Ice	09/09/18	64.2648	−15.8622	847	5.73	1.74	600^b^	n.a.	n.a.
February 2019 (winter)	IS19-1	Snæfellsjökull	Snow (c)	12/02/19	64.7818	−23.6654	170	6.28	10.20	11550^b^	1.00	70.00
	IS19-2	Langjökull	Snow (c)	13/02/19	64.6294	−20.4879	1,264	5.70	11.68	3150^c^	−5.80	52.50
	IS19-3	Langjökull	Snow (d)	13/02/19	64.6294	−20.4879	1,264	5.50	10.20	5700^c^	−5.80	52.50
	IS19-5	Skaftfella-jökull	Snow (d)	16/02/19	64.0312	−16.9281	117	7.68	51.76	1050^c^	0.90	40.60
	IS19-6	Skaftfella-jökull	Ice	16/02/19	64.0312	−16.9281	117	7.49	5.54	1045^c^	0.90	40.60
	IS19-7	Sólheima-jökull	Snow (d)	17/02/19	63.5376	−19.3397	201	6.10	3.29	3515^c^	4.6	54.40
	IS19-8	Sólheima-jökull	Ice	17/02/19	63.5376	−19.3397	201	6.52	6.71	2150^c^	4.6	54.40
August 2019 (summer)	IS19-10	Snæfellsjökull	Snow (d)	01/08/19	64.8141	−23.7514	947	6.33	2.34	550^b^	13.60	n.a.
	IS19-11	Snæfellsjökull	Ice	01/08/19	64.8141	−23.7503	940	5.69	1.63	500^b^	13.10	n.a.
	IS19-12	Snæfellsjökull	Snow–ice Interface	01/08/19	64.8145	−23.7492	919	4.92	2.70	300^b^	13.10	n.a.
	IS19-13	Langjökull	Snow (d)	02/08/19	64.6320	−20.5078	1,145	5.55	1.93	500^b^	5.40	n.a.
	IS19-14	Langjökull	Ice	01/08/19	64.6347	−20.5385	961	5.73	1.42	250^b^	7.40	n.a.

n.a., not analyzed; (c) clean; and (d) dirty.

a ~250 mg.

b Full 45 mm filter.

c Half 45 mm filter.

All samples were returned to the field laboratory, thawed at room temperature, and immediately processed once fully melted. For dissolved inorganic ions, sample aliquots were filtered through 0.2 μm polycarbonate filters into either acid-washed Nalgene® bottles (for cations, acidified upon return to the home institution with ultra-pure nitric acid), or into 15 ml Falcon® tubes (for anions). Samples for DOC analysis were filtered using acid-cleaned glass syringes, 0.7 μm ashed glass fiber filters (GFF), and acid-washed metal filtration units, and the solutions were filtered directly into acid-washed, ashed 40 ml amber vials sealed with an acid-cleaned Teflon septa. For solid particulate bulk carbon, nitrogen, and isotope analysis, we filtered between 0.5 and ~20 L of melted snow and ice through 0.7 μm ashed GFF filters. Resulting solids were transferred with ethanol-sterilized flat forceps into ashed glass vials. Upon return to the home laboratory, these solids were freeze-dried and milled with a ball mill (Retsch MM2000). For DNA analysis, between 0.05 and ~12 L (exact volumes in Table 1) of melted snow or ice, depending on season and particle load, were filtered through single use, sterile, 0.2 μm cellulose nitrate filters (Thermo Scientific Nalgene). Filters were removed from the units, folded with sterile forceps, and transferred, into sterile 5 ml cryotubes that were returned to the home laboratory in a cryo-shipper (filled with liquid nitrogen) and subsequently stored at −80°C, until extraction and analysis. A separate 15 ml Falcon® tube was filled with a sample preserved with glutaraldehyde (final concentration 2%) and returned to the home laboratory for microscopic observations.

### Dissolved and Particulates Analyses

The concentrations of dissolved inorganic major, minor, and trace elements in snow and ice samples were analyzed by inductively coupled plasma mass spectrometry (ICP-MS; Thermo Fisher iCAPQc) following methods described in McCutcheon et al. (2021). The precision of the analyses varied between 2% and 5%. Inorganic anions were analyzed by ion chromatography (IC) using conductivity detection (ICS 3000, Dionex). DOC was analyzed by LC-OCD (liquid chromatography—organic carbon detector) where organic carbon was quantified by IR detection of released CO_2_ after UV photo-oxidation (185 nm) in a Gräntzel thin-film reactor. Detailed descriptions of the latter two methods have been published elsewhere (Regenspurg et al., 2018).

Fine-milled particulate samples were analyzed for total carbon (TC), total nitrogen (TN), and carbon isotopic composition (δ^13^C) of TC using a Carlo Erba NC-2500 elemental analyzer coupled to a Finnigan DELTAplusXL isotope ratio mass spectrometer following approaches described previously in Lutz et al. (2016). Reproducibility was better than 0.1 wt % C, 0.01 wt % N, and <0.1‰ δ^13^C based on repeatedly measured standards.

### Microscopy

Glutaraldehyde fixed sample aliquots were filtered through 0.2 μm polycarbonate filters that were subsequently stained with 4′,6′-diamidino-2-phenylindole (DAPI; 1 μg ml^−1^). Slides with filter sections were examined under a fluorescent microscope (Leica DM 2000) and images acquired with a DFC 420C camera and a FI/RH (Leica) filter system by following Winkel et al. (2018).

### DNA Extraction, PCR Amplification, and Sequencing

DNA extractions were performed using the DNeasy PowerSoil Kit (QIAgen) according to the manufacturer’s protocol. DNA was eluted in 50 μl ultra-pure water. Extracted DNA concentration was measured on a Qubit 3.0 (Invitrogen) with the broad-range dsDNA kit (Invitrogen). Extracted DNA was amplified with bacterial primers for the 16S rRNA gene S-D-Bact-0341-b-S-17 (5′-CCTACGGGNGGCWGCAG-3′) and S-D-Bact-0785-a-A-21 (5′-GACTACHVGGGTATCTAATCC-3′; Herlemann et al., 2011); eukaryotic primers for the 18S rRNA gene 528F (5′-GCGGTAATTCCAGCTCCAA-3′) and 706R (5′-AATCCRAGAATTTCACCTCT-3′; Cheung et al., 2010), and the internal transcribed spacer 2 (ITS2) gene primers 5.8SbF (5′-GATGAAGAACGCAGCG-3′; Mikhailyuk et al., 2008) and ITS4R (5′-TCCTCCGCTTATTGATATGC-3′; White et al., 1990). Primers were tagged with Illumina adapter sequences. PCR was performed using KAPA HiFi HotStart ready mix (Roche) and the following PCR conditions: initial denaturation at 95°C for 3 min, 25 cycles (except samples collected in winter 2019 which were run for 35 cycles) of denaturation at 95°C for 30 s, annealing at 55°C for 30 s and elongation at 72°C for 30 s. Final elongation was run at 72°C for 5 min. All PCR runs were carried out in 25 μl reaction volumes. Amplified PCR products were sent to the Bristol Genomics Facility (United Kingdom), where they were barcoded with the Nextera XT Index kit. Pooled products were sequenced on the Illumina MiSeq platform using the V3 paired-end 2 × 300 bp chemistry.

### Bioinformatics

Raw sequences were first quality checked with the FastQC program (Andrews, 2010) for quality and read length. All three targeted marker genes (16S rRNA, 18S rRNA, and ITS2) were analyzed with the DADA2 R package (Callahan et al., 2016). Documentation of the marker gene analysis pipelines is publicly available in the project’s GitHub repository.^1^ Taxonomy was assigned for 16S and 18S rRNA gene sequences using the SILVA database (Release 132; Quast et al., 2012), while ITS2 gene sequences were assigned using the UNITE database (version 8.0; UNITE Community, 2019). The most abundant ASVs were manually blasted against the NCBI database to cross-correlate results and obtain detailed taxonomic information. Detailed sequencing statistics including the number of sequences retained at each step as well as final sequences retrained for each sample can be found in Supplementary Table S1. Note that recently the glacier ice algae *Mesotaenium* has been renamed (now *Ancylonema alaskana*; Procházková et al., 2021) as have several bacterial phyla (e.g., *Proteobacteria* are now *Pseudomonadota; Bacteriodetes* are now *Bacteriodota*; Oren and Garrity, 2021). Nevertheless, in the current contribution, we used the original names, because at the time of data evaluations the notations in the databases used had not yet been updated.

In our low biomass winter season (February 2019) samples, we detected many known bacterial contaminants that we could show derived from our extraction kits and those were removed from the further analyses of amplicon sequence variants (ASVs; Callahan et al., 2017) as suggested by Salter et al. (2014); Glassing et al. (2016). We retained sequences that have been previously documented as dominant in snow and ice environments (Lutz et al., 2015a; Perini et al., 2019) including *Polaromonas*, *Sediminibacterium*, *Pedobacter*, *Sphingomonas*, *Methylobacterium*, *Deinococcus*, and *Novosphingobium*.

### Statistical Analyses

Multivariate statistics were applied to environmental and sequencing data using with PAST v4 software (Hammer et al., 2001). Environmental parameters for principal component analysis (PCA) were log-transformed prior to statistical analyses, with the exception of pH according to Gobet et al. (2012). PCA was performed using an Euclidean distance matrix run with 1,000 iterations and PERMANOVA was used to test the difference between summer and winter samples. Sequencing data were Hellinger transformed (Legendre and Gallagher, 2001) prior to non-metric multidimensional scaling (NMDS) and hierarchical clustering. Diversity indices have been calculated on total ASVs with the implemented function of the PAST software.

### Data Deposition

Marker gene sequences of the snow and ice samples are deposited in the NCBI Sequence Read Archive (SRA) under BioProject ID# PRJNA657180, under the accession numbers SRR12490806-SRR12490826 for 16S rRNA and 18S rRNA gene sequences, and SRR12589324-SRR12589336 for ITS2 gene sequences.

## Results

### Geochemical Characteristics

A total of 20 winter and summer snow and bare ice samples were collected in 2018 and 2019 from 5 different glaciers and ice sheets in Iceland (Figure 1; Table 1).

**Figure 2:**
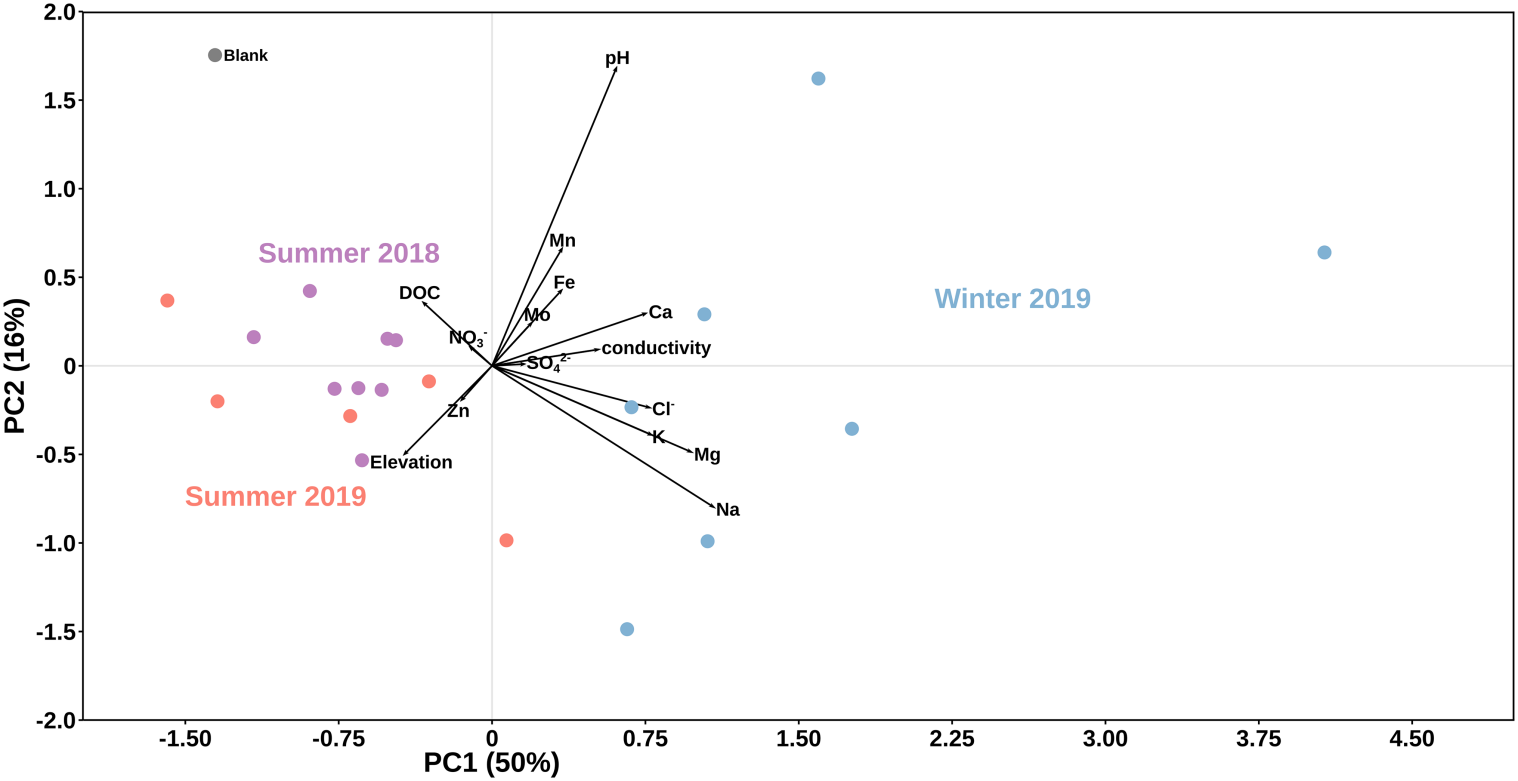
Principal component analysis of data by season. Black vectors indicate environmental/geochemical parameters while differently colored dots represent season of sample collection: purple for summer 2018, light blue for winter 2019, and light red for summer 2019. A field blank of filtered distilled water is shown in grey.

Overall, we found significant differences in geochemical signals and physical parameters (PERMANOVA, *p* = 0.0001) between summer and winter samples (Table 1; Figure 2). For example, conductivity values in winter (3.29–11.68 μs cm^−1^) were roughly double those in the summer samples (1.42–5.93 μs cm^−1^; Table 1), while pH values showed no seasonal trends (Figure 2). Comparison of the main dissolved ion concentrations revealed that snow had on average higher concentrations of sea-salt derived ions (Na, Cl, Mg, and K, Supplementary Figure S2 lower right quadrant), while ice contained higher concentrations of mineral-derived elements (e. g., Ca, Mn and Fe; Supplementary Figure S2, upper right quadrant). The major dissolved anions (Cl^−^, NO_3_^−^, and SO_4_^2−^) varied dramatically seasonally (Table 2). Chloride was detected in all samples, although in the winter samples it was up to many orders of magnitude higher than in the summer samples. Nitrate and sulfate were below detection limits (50 ppb) in all samples collected during summer [with the exception of measurable nitrate in the Langjökull pro-glacial outflow water sample (IS18-6)], but were detectable in the winter samples. Notably, sulfate concentrations in the winter snow samples were up to 10 times higher than in the winter ice samples. DOC was variable in all surface snow and ice samples, but overall, the summer 2018 samples were characterized by up to 20 times higher concentrations of DOC compared to both the winter 2018 and the summer 2019 samples. Finally, trace elements Fe, Mo, Mn, and in part Zn, which are crucial for metabolic purposes, varied seasonally and by habitat, with higher concentrations in the winter snow samples compared to summer snow and winter and summer ice samples (Table 2). Most other elements showed no seasonal trends. Comparing these parameters by location and by habitat (snow vs. ice) showed that Skaftafellsjökull samples were separated from all other sampling locations (Supplementary Figure S1), but that the ice and snow from Skaftafellsjökull also plotted distantly from each other on a PCA plot (Supplementary Figures S1, S2). The other snow–ice sample (winter pairs from Sólheimajökull and summer pairs from Langjökull and Snæfellsjökull) all plotted closer to each other compared to Skaftafellsjökull. Also, of note is that sample IS19-12, which is a red snow sample from the snow–ice surface interface from Snæfellsjökull plotted apart from all other samples in all PCA plots (Supplementary Figures S1, S2; Figure 1 for the snow–ice interface image, bottom left).

**Table 2:** Geochemical measurements of organic and inorganic species in aqueous phase.

Season	Sample ID	Location	Habitat	Cl^−^	NO_3_^−^	SO_4_^2−^	Na	K	Mg	Ca	Fe	Mn	Mo	Zn	DOC
September 2018 (summer)	IS18-1	Snæfellsjökull	Ice	60	bdl	bdl	bdl	4	bdl	bdl	<1	0.3	bdl	0.7	1.1
	IS18-2	Snæfellsjökull	Snow (d)	394	bdl	bdl	173	11	7	16	3	0.4	bdl	0.3	4.6
	IS18-3	Langjökull	Ice	54	bdl	bdl	bdl	bdl	bdl	bdl	1	0.7	bdl	0.6	1.8
	IS18-4	Langjökull	Snow–ice Interface	231	bdl	bdl	106	28	15	26	3	1.4	bdl	5.1	11.3
	IS18-5	Langjökull	Snow (d)	194	bdl	bdl	56	5	bdl	bdl	<1	0.2	bdl	0.3	0.7
	IS18-6	Langjökull	Pro-glacial water	197	505	bdl	63	3	21	37	3	0.6	bdl	3.8	20.5
	IS18-7	Vatnajökull	Snow (d)	205	bdl	bdl	49	5	5	bdl	3	0.4	bdl	3.4	16.5
	IS18-8	Vatnajökull	Ice	54	bdl	bdl	bdl	bdl	bdl	bdl	1	0.1	bdl	3.2	0.8
	IS19-1	Snæfellsjökull	Snow (c)	2,483	120	395	1,580	54	174	63	<1	2.0	5.23	2.6	0.4
February 2019 (winter)	IS19-2	Langjökull	Snow (c)	1,955	bdl	269	1,267	35	131	38	<1	bdl	1.26	1.5	0.5
	IS19-3	Langjökull	Snow (d)	1,675	bdl	268	1,077	37	99	36	1	<0.1	2.13	2.4	0.5
	IS19-5	Skaftfellajökull	Snow (d)	14,013	110	1,999	8,599	280	890	886	12	2.5	10.48	0.9	0.7
	IS19-6	Skaftfellajökull	Ice	302	480	118	330	22	59	931	17	2.1	2.94	0.4	0.5
	IS19-7	Sólheimajökull	Snow (d)	928	bdl	121	665	19	49	36	2	1.1	1.05	1.4	0.4
	IS19-8	Sólheimajökull	Ice	754	bdl	bdl	585	34	41	41	6	1.3	2.26	3.7	0.5
August 2019 (summer)	IS19-10	Snæfellsjökull	Snow (d)	397	bdl	bdl	301	11	11	12	<1	0.4	1.16	1.2	0.4
	IS19-11	Snæfellsjökull	Ice	bdl	bdl	bdl	44	5	4	7	1	0.2	2.50	1.7	0.6
	IS19-12	Snæfellsjökull	Snow–ice Interface	352	bdl	bdl	245	49	49	20	<1	0.6	0.96	1.4	0.6
	IS19-13	Langjökull	Snow (d)	282	bdl	bdl	184	6	6	24	<1	1.3	bdl	0.7	0.3
	IS19-14	Langjökull	Ice	bdl	bdl	bdl	9	3	3	15	2	0.6	bdl	1.3	0.6

(c) clean, (d) dirty, DOC is given in ppm, Cl^−^, NO_3_^−^, SO_4_^2−^, Fe, Na, K, Mg, Ca, Mn, Zn are given in ppb, Mo is given in ppt; bdl below detection limit; (c) clean, (d) dirty, LOD’s for ICP-MS: Mg, Ca = 3 ppb; K, Na, 1 ppb, Fe = 0.5 ppb, Mn, Zn = 0.02 ppb, Mo = 0.3 ppt; LOD’s for IC: Cl^−^, NO_3_^−^, SO_4_^2−^, DOC = 0.05 ppm.

The difference between winter and summer samples was also mirrored in the total organic carbon (TOC %, Table 3) contents and isotope signature of the winter vs. the summer samples. The summer solid particulate samples contained far higher TC contents ranging from BDL to an exceptionally high value of 6.8% in the snow–ice interface sample IS19-12. In contrast, all winter samples were characterized by low TC values (max 0.41%, Table 3) that were on average one order of magnitude lower compared to the summer samples. In addition, all TN values in the winter samples were below detection limit of our analyses (<0.1%).

**Table 3:** Bulk measurements of carbon, nitrogen and carbon stable isotopes based on dry weight. C/N ratios calculated from the TC and TN values.

Season	Sample ID	Location	Habitat	TC (%)	TN (%)	^13^C (‰)	^15^N (‰)	C/N
September 2018 (summer)	IS18-1	Snæfellsjökull	Ice	3.09	0.24	−26.3	n.a.	12.88
	IS18-2	Snæfellsjökull	Snow (d)	2.90	0.26	−28.1	n.a.	11.15
	IS18-3	Langjökull	Ice	0.35	bdl	−26.6	n.a.	n.a.
	IS18-4	Langjökull	Snow–ice Interface	n.a.	n.a.	n.a.	n.a.	n.a.
	IS18-5	Langjökull	Snow (d)	2.89	0.28	−29.1	n.a.	10.32
	IS18-6	Langjökull	Pro-glacial water	n.a.	n.a.	n.a.	n.a.	n.a.
	IS18-7	Vatnajökull	Snow (d)	2.84	0.28	−30.2	n.a.	10.14
	IS18-8	Vatnajökull	Ice	0.81	0.14	−26.6	n.a.	5.79
February 2019 (winter)	IS19-1	Snæfellsjökull	Snow (c)	n.a.	n.a.	n.a.	n.a.	n.a.
	IS19-2	Langjökull	Snow (c)	n.a.	n.a.	n.a.	n.a.	n.a.
	IS19-3	Langjökull	Snow (d)	n.a.	n.a.	n.a.	n.a.	n.a.
	IS19-5b	Skaftfellajökull	Snow (d)	0.25	bdl	−25.5	n.a.	n.a.
	IS19-5c	Skaftfellajökull	Snow (d)	0.09	bdl	−21.0	n.a.	n.d.
	IS19-6a	Skaftfellajökull	Ice	0.24	bdl	−24.9	n.a.	n.d.
	IS19-6b	Skaftfellajökull	Ice	0.21	bdl	−20.5	n.a.	n.d.
	IS19-6c	Skaftfellajökull	Ice	0.11	bdl	−22.0	n.a.	n.a.
	IS19-7a	Sólheimajökull	Snow (d)	0.23	bdl	−26.3	n.a.	n.a.
	IS19-7b	Sólheimajökull	Snow (d)	0.16	bdl	−25.3	n.a.	n.a.
	IS19-8	Sólheimajökull	Ice	0.41	bdl	−25.9	n.a.	n.d.
August 2019 (summer)	IS19-10	Snæfellsjökull	Snow (d)	2.92	0.28	−27.5	−6.3	10.43
	IS19-11	Snæfellsjökull	Ice	4.08	0.38	−26.3	−6.4	10.74
	IS19-12	Snæfellsjökull	Snow–ice Interface	6.80	0.86	−29.2	−9.9	7.91
	IS19-13	Langjökull	Snow (d)	0.33	bdl	−28.5	bdl	n.d.
	IS19-14	Langjökull	Ice	0.28	bdl	−26.4	bdl	n.d.

(c) clean; (d) dirty; bdl, below detection limit; n.d., not determined; and n.a., not analyzed.

In terms of the carbon isotopic signatures, we found that the winter samples were characterized by heavier stable carbon isotope signals (summer average δ^13^C > ~ −27‰; and winter average δ^13^C < ~ −25‰). Differences were also noticeable between ice and snow pairs from the same glacier and sampled at the same time in the summer samples, with ice having heavier δ^13^C (Table 3). Winter samples showed similar values for both ice and snow. Because all winter sample had BDL TN contents, C/N vs. δ^13^C values could only be plotted for the summer samples (Supplementary Figure S5; Table 3).

### Habitat and Seasonal Variation in Microbial Community Composition

#### Eukaryotic Communities

**Figure 3:**
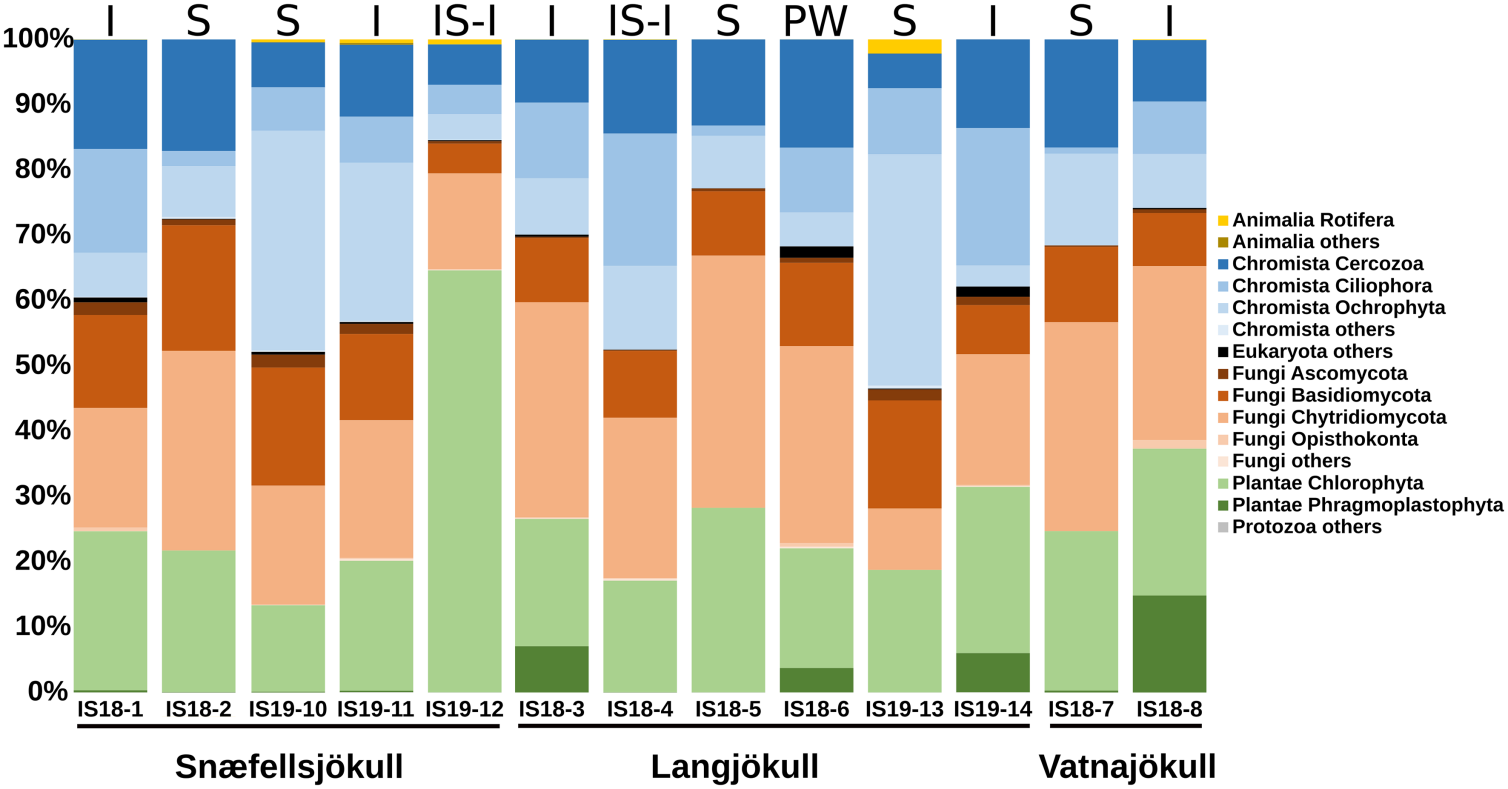
Stacked bar plots showing 18S rRNA gene relative abundance at the phylum level. Samples are ordered chronologically for the each glacier. Letters above bars indicate habitat with I = ice, S, snow; SI-I, snow–ice interface; and PW, pro-glacial water and numbers below bars indicate sample number (Table 1). Only summer samples are shown because 18S rRNA amplification of the winter 2019 samples did not yield a product.

We targeted the 18S rRNA gene and ITS2 genes for amplification and sequencing of eukaryotes, but only had successful amplification of these genes in samples collected during summer. Overall, *Fungi*, *Plantae*, and *Chromista* dominated the eukaryotic community in both our snow and bare ice samples (Figure 3). The dominant fungi phyla *Basidiomycota* and *Chytridiomycota* made up between 19% and 50% of reads in all samples. The *Chromista* were dominated by *Cercozoa* (5%–17%), *Ciliophora* (1%–21%), and *Ochrophyta* (5%–35%). The latter group contained snow algae of the genus *Hydrurus*, commonly found in “yellow snow” (Remias et al., 2013). The *Plantae* consisted of the two phyla *Chlorophyta* (13%–65%) and *Phragmoplastophyta* (<1%–15%; Figure 3). The *Chlorophyta* include the typical snow algae of the genera *Chloromonas*, *Chlainomonas*, and *Sanguina*, which cause the often observed red and green snow blooms (Lutz et al., 2016; Remias et al., 2016; Engstrom et al., 2020). The dominant glacier ice algae in our samples were *Phragmoplastophyta*, which include the well-known glacier ice algae of the genera *Mesotaenium* and *Ancylonema* (Remias et al., 2009, 2012).

**Figure 4:**
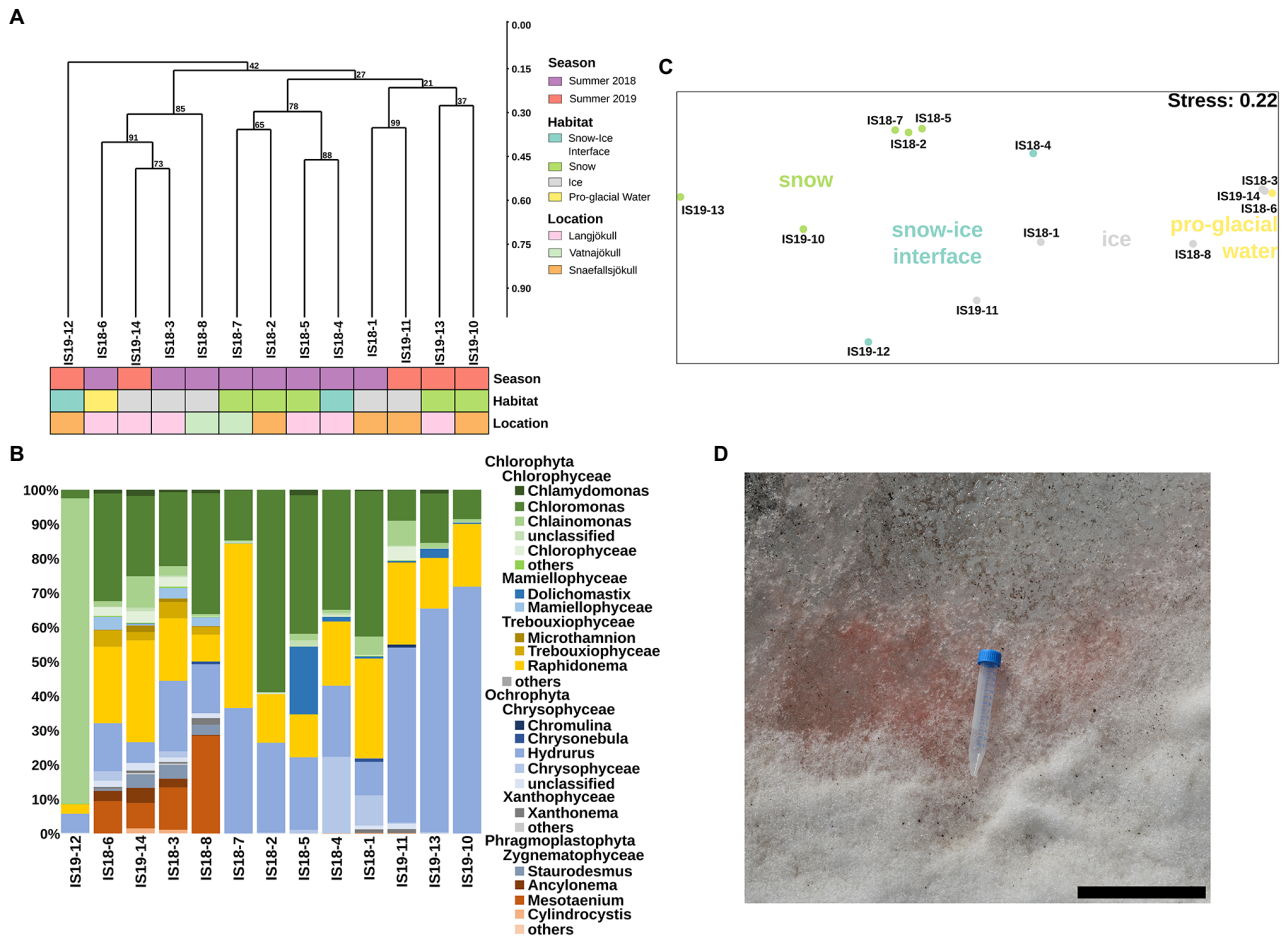
Algae community structure based on 18S rRNA gene abundance. **(A)** Hierarchical clustering of 18S rRNA algae ASVs using Bray–Curtis and 1,000 iterations underlain by colored boxes that show season (red, purple), location (pink, orange, cyan), and habitat (light blue, green, gray, yellow). **(B)** Stacked barplots showing 18S rRNA gene ASVs relative abundance at the genus level with bars ordered according to hierarchical clustering. **(C)** NMDS plot of 18S rRNA gene ASVs using Bray–Curtis dissimilarities of Hellinger transformed ASVs with sampling sites color coded according to habitats as shown in legend in **(A)**. **(D)** Close up image of field site IS19-12 showing a dominance of red snow with the 15 ml Falcon tube as size reference and the scale bar representing 12 cm.

We extracted algal ASVs and clustered them using Bray–Curtis dissimilarities, revealing that samples were mainly clustering according to differences in habitat—with the exception of the snow–ice interface sample IS19-12 (Figure 4A) and ice samples from Snæfellsjökull (IS18-1 and IS19-11). In sample IS19-12, more than 90% of the reads belonged to the genus *Chlainomonas* (Figure 4B), with its typical red xanthophyll rich pigmentation that causes the dark red color of this sample (Figure 4D). One cluster (IS18-6, IS19-14, IS18-3, IS18-8), which contained only ice samples and the pro-glacial water sample, had high numbers of the genera *Mesotaenium* and *Ancylonema* (Figure 4B). Summer ice samples from Snæfellsjökull (IS18-1 and IS19-11) contained negligible proportions of glacier ice algae and clustered with snow from the other summer samples (Figure 4A). *Chloromonas* and *Hydrurus* and the permafrost/soil algae *Raphidonema* were dominant in all snow samples and in the snow–ice interface sample IS18-4 (Figure 4B). Beta diversity analysis *via* NMDS of all samples showed a separation between snow and ice samples, with snow–ice interface samples (IS18-4 and IS19-12) plotting in between. The pro-glacial water sample clustered with the ice samples (Figure 4C).

In all summer samples, sequencing of the ITS2 gene showed a dominance of *Chloromonas* across all habitats and locations, with the exception of the snow–ice interface sample IS19-12, which was dominated by *Chlainomonas* (Supplementary Figure S3B). This result mirrors our 18S rRNA gene data (Figure 4), but the sequencing of the ITS2 gene missed the glacier ice algae of the phylum *Phragmoplastophyta* that were present in our 18S rRNA data (Figure 4 vs. Supplementary Figure S3A). Contradictory to the 18S rRNA gene sequences (Figure 4), we found that in the ITS our samples were dominated by *Koliellaceae*/*Pseudochlorella* (Supplementary Figure S3B), but other dominant taxa such as *Hydrurus*, seen in the 18S rRNA data, were missing (Figure 4B).

#### Bacterial Communities

**Figure 5:**
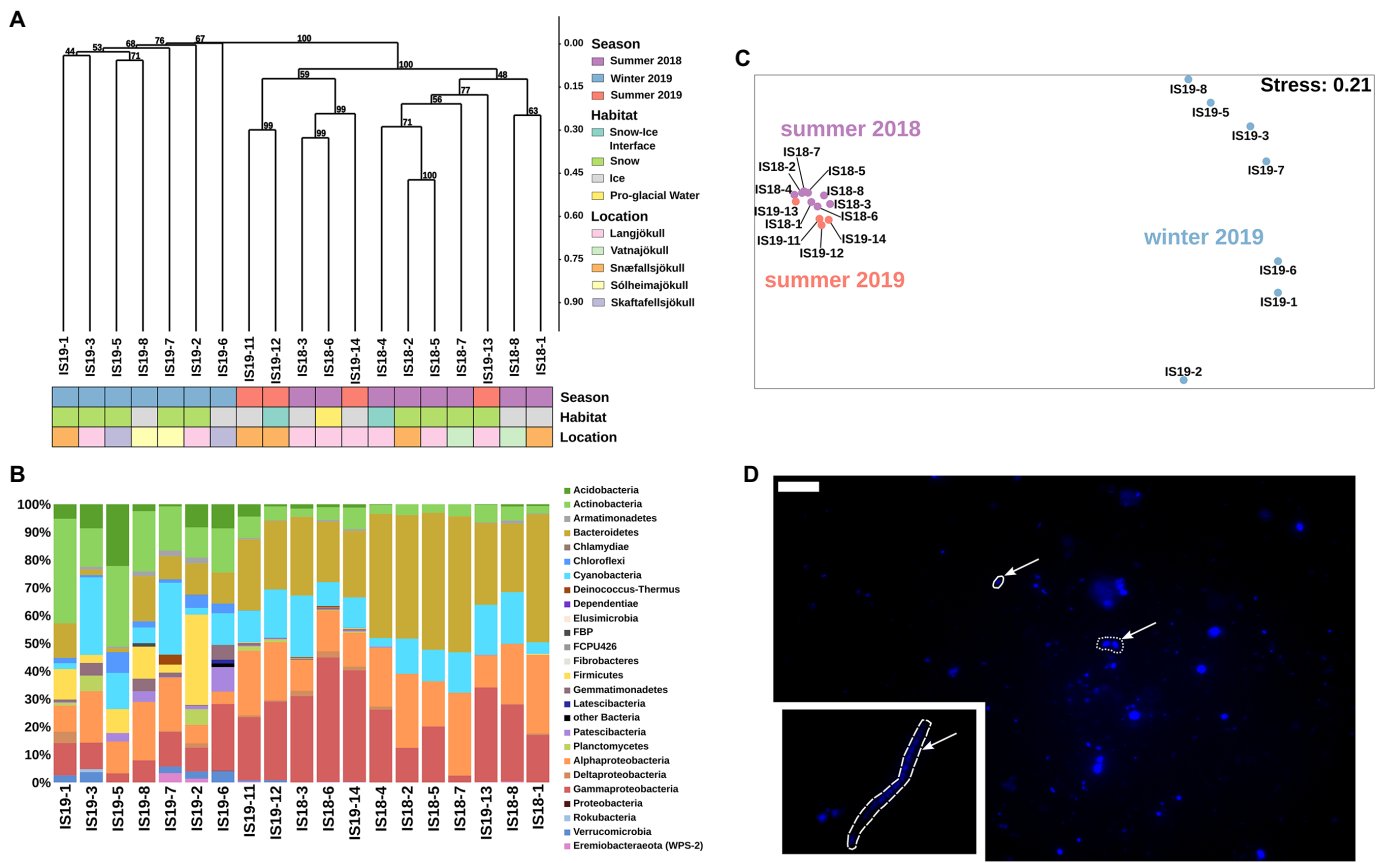
Bacterial community structure based on 16S rRNA gene abundances. **(A)** Hierarchical clustering of 16S rRNA algae ASVs using Bray–Curtis and 1,000 iterations underlain by colored boxes that show season (red, purple, blue), locations (pink, orange, cyan, light purple, light yellow), and habitats (light blue, green, gray, yellow). **(B)** Stacked barplots showing 16S rRNA gene ASVs relative abundances at the class level. Bars have been ordered according to hierarchical clustering. **(C)** NMDS plot of 16S rRNA gene ASVs using Bray–Curtis dissimilarities of Hellinger transformed ASVs with sampling sites color coded per season as shown in legend in **(A)**. **(D)** Microscopic images of DAPI-stained cells. Arrow points toward different cell morphologies: solid = rod, dotted = coccoid, and dashed = filamentous. Scale bar represents 10 μm. Direct imaging of DAPI-stained cells using epifluorescence microscopy revealed a range of microbial morphologies from cocci, and rod-shaped cells (**(D)**, arrows in main image) to filaments (**(D)**, inset).

Bacterial 16S rRNA genes were amplified and sequenced in all winter and summer samples and across all habitats. DNA concentrations were low or below Qubit detection levels (<0.01 μg ml^−1^) in all winter samples. Sequences were clustered using Bray–Curtis dissimilarities. We found that summer and winter bacterial communities were distinct from each other (Figure 5A). Bacterial communities in summer (2018 and 2019) were very similar to each other, only partially clustering by habitat type, while they clearly differed from winter communities (Figure 5).

Our summer samples were dominated by *Bacteriodetes* (22% and 49%), *Gammaproteobacteria* (2% and 45%), *Alphaproteobacteria* (12% and 30%), *Cyanobacteria* (4% and 26%), *Actinobacteria* (up to 8%), and *Acidobacteria* (up to 4%). The summer snow samples showed slightly lower proportions of *Gammaproteobacteria*, negligible *Acidobacteria* (Figure 5B), and had the lowest alpha diversity (Observed ASVs and Simpson index, Supplementary Figure S4). The summer ice samples clustered together with the snow–ice interface and the pro-glacial water samples and were characterized by low abundance taxa such as *Planctomycetes*, *Gemmatimonadetes*, *Firmicutes*, *Deinococcus-Thermus*, *Chloroflexi*, *Verrucomicrobia*, and *Eremiobacteraeota* (WPS-2). These were mostly absent in the summer snow samples cluster (Supplementary Figure S6). The winter samples generally had lower numbers of observed ASVs (~100), compared to the summer samples (between 50 and 500), and contained different taxa to the summer samples. Nevertheless, summer and winter snow and ice had similar Simpson indices (Supplementary Figure S4). In all winter samples, we saw an increased proportion of reads assigned to *Actinobacteria* (11%–38%), *Firmicutes* (up to 32%), *Acidobacteria* (1%–22%), *Chloroflexi* (1%–7%), *Verrucomicrobia* (up to 4%), and *Armatimondetes* (up to 2%); these phyla were almost exclusively found in our winter samples. Moreover, the proportion of reads for taxa that were dominant in the summer, such as *Bacteriodetes*, *Gammaproteobacteria*, and *Alphaproteobacteria* were on average 2.5-fold lower in the winter samples (Figure 5C). Some highly abundant taxa such as *Cyanobacteria* (more than 25% all ASVs in all samples with the exception of IS19-7 and IS19-3) showed similar abundances across both winter and summer. An NMDS plot using Bray–Curtis dissimilarities showed a separation between winter and summer samples: the summer samples clustered close to each other, while the winter samples were highly distinct (Figure 5C). Bacterial cells could be visualized by DAPI staining and showed a variety of different morphologies (Figure 5D).

Comparing winter and summer samples on an ASV level showed that they only share 30 ASVs, 7 of which belonging to chloroplast and mitochondria (Supplementary Table S2). The remaining 976 ASVs in our summer samples and 678 ASVs in our winter samples belonged to similar higher taxonomical levels (order to genus), but showed differences on ASV frequencies. For example, *Bacteriodetes* contained specific summer genera (*Paludibacter*, *Prolixibacteraceae*/BSV13, and WCHB1-32, *Sedminibacterium*, and *Lentimicrobiaceae*), whereas other genera (*Alloprevotella*, *Terrimonas*, and *Flavisolibacter*) only appeared in winter (Supplementary Figure S6). Similar patterns as for *Bacteriodetes* were found for *Actinobacteria*, *Cyanobacteria*, *Alphaproteobacteria*, and *Gammaproteobacteria* (Supplementary Table S2).

## Discussion

Our data reveal clear seasonal shifts in the microbial community with a link to the geochemical characteristics of the glacier surface environment.

The three dominant eukaryotic phyla found in summer snow and ice samples (*Chromista*, *Fungi*, and *Plantae*) have been found previously on Icelandic glaciers (Lutz et al., 2015a) and elsewhere including snow from the Rocky Mountains (United States; Havig and Hamilton, 2019) and cryoconite holes in Queen Maud Land, Antarctica (Lutz et al., 2019), however in lower relative abundance than we detected here. The glacier ice algae *Ancylonema*, *Mesotaenium*, and *Cylindrocystis* (Figure 4) are also typical of glacier ice samples, as determined by conventional microscopic analysis (Takeuchi, 2001; Takeuchi and Kohshima, 2004; Uetake et al., 2010; Remias et al., 2012; Williamson et al., 2018). However, so far, only two studies have documented a large dominance of *Ancylonema* and *Mesotaenium* in glacier surface ice samples using molecular techniques (Lutz et al., 2018; McCutcheon et al., 2021). The clade *Phragmoplastophyta*, which was present in our 18S rRNA data but not in our ITS2 data (pointing toward a potential PCR primer bias) has previously been shown to comprise the majority of algae inhabiting glacier ice (Takeuchi, 2001; Takeuchi and Kohshima, 2004; Uetake et al., 2010; Remias et al., 2012; Lutz et al., 2018; Williamson et al., 2018; McCutcheon et al., 2021).

The summer snow algal community in our samples largely reflects findings from other similar (i.e., glacier surface snow) environments. In particular, *Raphidonema* and *Hydrurus* sp. have been shown to be common on Icelandic glaciers (Lutz et al., 2015a). Interestingly, the red-colored summer snow–ice interface sample from Snæfellsjökull (IS19-12) was dominated by *Chlainomonas* and not by the previously reported *Chloromanas* and *Chlamydomonadaceae* (Lutz et al., 2015a). *Chlainomonas* has rarely been documented among snow algal communities (Novis, 2002b), and has previously been reported in few locations (Hoham, 1975; Novis, 2002a, b; Remias et al., 2016; Engstrom et al., 2020). The abundance and diversity of algae found in our summer samples was not mirrored in our winter samples; they were fully absent from genomic data from our winter samples. Eukaryotic ice and snow algae require liquid water to bloom (Lutz et al., 2014; Anesio et al., 2017), and the scarcity of water during winter likely prohibits or slows algal metabolism. Moreover, in several of our winter sampling locations (i.e., Snæfellsjökull and Langjökull) glacier ice algae may have been present in deeper ice layers below the snow cover that we could not reach in our sampling efforts. The presence of such layers representing algal communities “frozen-in” at the end of the previous melt season is not well established and should be the target of future studies. Also, it remains unclear why glacial ice algae *Ancylonema* and *Mesotaenium* are so under-represented on Icelandic glaciers in comparison to the Greenland Ice Sheet or other Arctic settings where they are often found to dominate the glacier ice algae community (Lutz et al., 2017, 2018).

Bacteria were detected in all samples, but notably, the community composition differed significantly between summer and winter. The winter bacterial community was similar to air samples from Antarctica (Archer et al., 2019) and the Austrian Alps (Els et al., 2020), and also to fresh early spring snow from Svalbard, Norway (Larose et al., 2010; Maccario et al., 2014), based on previous studies that analyzed the same marker gene (16S rRNA) or the entire metagenome. The bacterial communities present in our summer samples shared similarities with those communities characterized in previous studies of summer-time glacial ecology (Hell et al., 2013; Lutz et al., 2015b; Hamilton and Havig, 2017; Havig and Hamilton, 2019).

Heterotrophic groups such as *Bacteriodetes*, *Alphaproteobacteria*, and *Gammaproteobacteria* dominated in all summer samples and belong to known groups that have been identified in marine systems to degrade algae-derived polysaccharides (Ruff et al., 2014; Teeling et al., 2016). These heterotrophs express carbohydrate-active enzymes known as CAZymes (Lombard et al., 2014), such as glycoside hydrolases (GH) that are most likely involved in the degradation of algae exudates (e.g., polysaccharides; Teeling et al., 2016). *Cyanobacteria*, while previously shown to inhabit cryoconite holes on glaciers and ice sheets (Stibal et al., 2008; Edwards et al., 2011), ice surfaces (Yallop et al., 2012), and some snow (Lutz et al., 2015a, 2016), often classify as Chloroplast in 16S rRNA gene sequencing. However, in this case the majority of sequences classified as Chloroplast are likely derived from the high abundance of glacier ice algae in our algal rich summer samples.

The variability in microbial communities between winter and summer was mirrored in the geochemical signals measured in the snow and ice samples. High concentrations of DOC were associated with summer algal blooms. During summer, glacial meltwater exports significant quantities of DOC (Lawson et al., 2014; Musilova et al., 2017), while during winter, freezing conditions on the glacier surface likely prevent supraglacial DOC export (Hood et al., 2009; Singer et al., 2012). It has recently been shown that primary production by supraglacial algae and other microorganisms over a summer melt season leads to an increase in the bioavailability of the meltwater-exported DOC (Kellerman et al., 2020). The high DOC concentrations measured in our summer 2018 snow samples (between 4 and 20 ppm, Table 2) are at the upper-most range of what has previously been measured for glacial ice (Hood et al., 2015), exceeding typical glacial snowpack and glacial outflow concentrations (Singer et al., 2012; Stubbins et al., 2012; Yan et al., 2016), and are largely in line with measured DOC concentrations in green snowpacks (i.e., snow containing abundant *Chlorophyta*) in Svalbard (Lutz et al., 2015b), and in ice samples from Antarctica (Barker et al., 2006). We suggest that the relatively high DOC concentrations in our samples compared to others (Singer et al., 2012; Stubbins et al., 2012; Hood et al., 2015; Yan et al., 2016) could arise from (i) our sampling at the end of the summer season (i.e., ~mid-September 2018) compared to other studies sampling between May and August (Singer et al., 2012; Stubbins et al., 2012; Wright et al., 2013; Lutz et al., 2015a,b; Yan et al., 2016), and (ii) the relatively high nutrient loading (and therefore high primary productivity) experienced by Icelandic glaciers due to volcanic input (Bagnato et al., 2013; Galeczka et al., 2017).

The combined organic δ^13^C signature and the C/N ratios for the summer samples provide an indication of the origin of the organic material (Lamb et al., 2006). Summer DOC (Table 2) is likely strongly influenced by microbially derived organic matter, evidenced by low C/N ratios (<13) and low δ^13^C-values (← ~ −25‰), typical of freshwater values (Lamb et al., 2006; Spijkerman et al., 2012). Compared to other glaciers in Svalbard and Sweden, the C/N and δ^13^C characteristics of our summer samples were similar to previously reported green snow and dirty ice samples (Lutz et al., 2017).

We found seasonal differences in the major macro- and micronutrients that are relevant for catabolic and anabolic cell processes (Goldman et al., 2012), for co-factors for enzyme activity, and that are used in anti-freeze protection mechanisms (Garnham et al., 2011). The concentrations of major dissolved ions in all our snow and ice samples (Table 2), fell within the range of concentrations reported in previous studies for Iceland snow and ice samples (Lutz et al., 2015a; Galeczka et al., 2017). In most cases, the ion concentrations were higher during winter than summer regardless of habitat (i.e., bare ice or snow; Table 2). Low summer nutrient and trace elements concentrations (e.g., Na, K, Mg, Ca, SO_4_^2−^, NO^3−^, Mn, Mo, and Fe) correspond to the timing of algal blooms (i.e., summer). The availability of nitrogen and phosphorous are known to limit biological productivity of glacier surface ecosystems (Wolff, 2013; Williams et al., 2016; McCutcheon et al., 2021).

The high concentrations of NO_3_^2−^ detected in Snæfellsjökull, Skaftfellajökull, and Sólheimajökull winter snowpacks may arise from direct dry deposition of reactive inorganic nitrogen in snow (as observed in Svalbard; Kühnel et al., 2012, 2013; Björkman et al., 2013), biological nitrogen fixation (for example, by *Cyano*- and *Proteobacteria*; Telling et al., 2011), by cycling of organic nitrogen (Holland et al., 2019) or microbial necromass from glacial or pro-glacial environments (Bradley et al., 2016). While some chemical measurements point toward nitrification (Telling et al., 2014; Wadham et al., 2016), only one study directly identified putatively involved microorganisms (Segawa et al., 2014). Ammonia-oxidizing microorganisms such as *Nitrosomonadaceae* (Prosser et al., 2014) were present in our ice samples but were rarely found in our snow samples. Sulfate, in both our summer and winter samples, was likely derived from atmospheric deposition as sea-spray (e.g., Lutz et al., 2015a, 2016), or aerosol sulfate.

## Conclusion and Outlook

Our data reveal seasonal characteristics of glacier surface ecosystems. We show a clear shift in the microbial community structure and corresponding geochemical environmental characteristics from the summer to the winter season.

Summer periods were characterized by a higher availability of microbially derived DOC and low nutrient concentrations, whereas winter was characterized by lower DOC and high nutrient concentrations.

The summer eukaryotic algae inhabiting the glacier surface ice and snow were distinct and were closely associated with a diverse community of heterotrophic bacteria likely relying on algal-delivered DOC. Winter microbial communities contained bacteria resembling communities typically found in air and snow and lacked eukaryotic algae.

The low abundance of glacial ice algae *Ancylonema* and *Mesotaenium* during summer in Iceland in comparison to the dominance in Greenlandic glaciers (Lutz et al., 2018; McCutcheon et al., 2021) is still unclear and should be further explored. Additionally, future work should identify the seasonal dynamics of glacier ice algae—specially to clarify whether their apparent absence in winter samples (as suggested in this study) is a consequence of sampling (i.e., algae may be present in deeper snow/ice interface layers) or is due to other ecological factors that drive algal distribution and abundance seasonally. In addition, summer and winter are punctuated by spring thaw and autumn freezing periods, which must also be studied to fully close the remaining gaps in understanding year-round characteristics of glacial ecosystems.

## Data Availability Statement

The datasets presented in this study can be found in online repositories. The names of the repository/repositories and accession number(s) can be found in the article/Supplementary Material.

## Author Contributions

MW and LGB designed the study. MW, CBT, and RM carried out laboratory work. MW and CBT performed bioinformatic analysis. MW wrote the first draft of the manuscript. All authors contributed to the sample collection in Iceland and contributed guidance and edits to the final manuscript preparation. All authors contributed to the article and approved the submitted version.

## Funding

MW, RM, CBT, and LGB were funded through a Helmholtz Recruiting Initiative grant no. I-044-16-0. During the writing of this manuscript, LGB was also supported through an ERC Synergy grant (“DeepPurple” grant # 856416) from the European Research Council (ERC) funded under the European Union’s Horizon 2020 research and innovation program. JAB was part funded by an Alexander von Humboldt Foundation postdoctoral fellowship and a UK NERC grant (NE/T010967/1). The open access publication of this paper was supported within the funding program “Open Access Publikationskosten” Deutsche Forschungsgemeinschaft (DFG, German ResearchFoundation)—project number 491075472.

## Conflict of Interest

The authors declare that the research was conducted in the absence of any commercial or financial relationships that could be construed as a potential conflict of interest.

## Publisher’s Note

All claims expressed in this article are solely those of the authors and do not necessarily represent those of their affiliated organizations, or those of the publisher, the editors and the reviewers. Any product that may be evaluated in this article, or claim that may be made by its manufacturer, is not guaranteed or endorsed by the publisher.

## Footnotes

^1^ https://doi.org/10.5281/zenodo.5897244
