# Supplementary material for: Seasonality of Glacial Snow and Ice Microbial Communities
Source: Front Microbiol. 2022 May 16;13:876848. doi: 10.3389/fmicb.2022.876848 (PMC9149292; doi:10.3389/fmicb.2022.876848)
Supplement: Supplementary file 1 [file Table_1.docx]

**Seasonality of glacial snow and ice microbial communities**

Matthias Winkel^1,2,*^, Christopher B. Trivedi^1^, Rey Mourot^1^, James A. Bradley^1,3^, Andrea Vieth-Hillebrand^1^, Liane G Benning^1,4^

^1^GFZ German Research Centre for Geosciences, Helmholtz Centre for Geosciences, Germany, Potsdam

^2^New address: BfR, Federal Institute for Risk Assessment, 5.1 Analytic and Toxikogenomics, Berlin, Germany

^3^Queen Mary University London, London, UK

^4^Department of Earth Sciences, Free University of Berlin, 12249, Berlin, Germany

*Corresponding author: Matthias Winkel, [mwinkel1982@gmail.com](mailto:mwinkel1982@gmail.com) and Liane G. Benning, [benning@gfz-potsdam.de](mailto:benning@gfz-potsdam.de)

**Table S1| Sequencing statistics of analysed genes showing raw, quality filtered and denoised/chimera checked/contamination filtered sequences.**

| Season | Sample ID | Location | Habitat | 16S rRNA | | | 18S rRNA | | | ITS2 | | |  |
| --- | --- | --- | --- | --- | --- | --- | --- | --- | --- | --- | --- | --- | --- |
|  |  |  |  | Raw | Quality filtered | Denoised, Chimera checked, Cont. filtered | Raw | Quality filtered | Denoised, Chimera checked, Cont. filtered | Raw | Quality filtered | Denoised, Chimera checked, Cont. filtered |  |
| **September 2018**  **(summer)** | IS18-1 | Snæfellsjökull | Ice | 133,140 | 88,604 | 62,469 | 256,581 | 214,594 | 184,564 | 192,071 | 143,861 | 109,137 |  |
|  | IS18-2 | Snæfellsjökull | Snow (d) | 182,575 | 131,065 | 110,178 | 303,343 | 253,346 | 211,227 | 288,435 | 157,606 | 144,006 |  |
|  | IS18-3 | Langjökull | Ice | 207,022 | 141,647 | 112,140 | 243,453 | 188,471 | 162,057 | 192,429 | 138,852 | 113,274 |  |
|  | IS18-4 | Langjökull | Snow-ice  Interface | 191,573 | 132,248 | 104,248 | 395,575 | 329,687 | 277,817 | 182,378 | 135,794 | 107,580 |  |
|  | IS18-5 | Langjökull | Snow (d) | 216,588 | 151,058 | 131,789 | 391,876 | 322,000 | 271,620 | 360,163 | 184,891 | 160,587 |  |
|  | IS18-6 | Langjökull | Pro-glacial water | 198,831 | 137,941 | 102,490 | 366,767 | 292,387 | 261,090 | n.d. | n.d. | n.d. |  |
|  | IS18-7 | Vatnajökull | Snow (d) | 188,059 | 134,750 | 98,760 | 282,613 | 231,045 | 196,076 | 251,727 | 141,154 | 124,904 |  |
|  | IS18-8 | Vatnajökull | Ice | 232,370 | 165,442 | 114,407 | 331,497 | 268,096 | 227,952 | 245,027 | 174,053 | 141,909 |  |
|  | IS18-L |  | Lab blank | 16,241 | 10,614 | 1,595 | 42,059 | 32,813 | 17,464 | 557 | 87 | 16 |  |
| **February 2019**  **(winter)** | IS19-1 | Snæfellsjökull | Snow (c) | 160,571 | 137,691 | 105,123 | n.d. | n.d. | n.d. | n.d. | n.d. | n.d. |  |
|  | IS19-2 | Langjökull | Snow (c) | 155,353 | 131,233 | 97,281 | n.d. | n.d. | n.d. | n.d. | n.d. | n.d. |  |
|  | IS19-3 | Langjökull | Snow (d) | 152,088 | 129,086 | 48,520 | n.d. | n.d. | n.d. | n.d. | n.d. | n.d. |  |
|  | IS19-5 | Skaftfellajökull | Snow (d) | 165,779 | 140,586 | 38,902 | n.d. | n.d. | n.d. | n.d. | n.d. | n.d. |  |
|  | IS19-6 | Skaftfellajökull | Ice | 121,269 | 103,843 | 63,695 | n.d. | n.d. | n.d. | n.d. | n.d. | n.d. |  |
|  | IS19-7 | Sólheimajökull | Snow (d) | 154,676 | 130,073 | 44,847 | n.d. | n.d. | n.d. | n.d. | n.d. | n.d. |  |
|  | IS19-8 | Sólheimajökull | Ice | 123,179 | 105.389 | 23,193 | n.d. | n.d. | n.d. | n.d. | n.d. | n.d. |  |
| **August 2019**  **(summer)** | IS19-10 | Snæfellsjökull | Snow (d) | n.a. | n.a. | n.a. | 267,353 | 240,217 | 203,015 | 146,746 | 106,728 | 72,337 |  |
|  | IS19-11 | Snæfellsjökull | Ice | 125,062 | 106,436 | 93,601 | 210,286 | 188,743 | 157,229 | 222,494 | 158,925 | 118,910 |  |
|  | IS19-12 | Snæfellsjökull | Snow-ice  Interface | 84,154 | 72,685 | 63,134 | 213,050 | 194,376 | 154,466 | 164,723 | 117,366 | 91,784 |  |
|  | IS19-13 | Langjökull | Snow (d) | 121,159 | 103,153 | 91,810 | 214,676 | 195,568 | 169,541 | 177,368 | 133,911 | 109,030 |  |
|  | IS19-14 | Langjökull | Ice | 121,078 | 102,416 | 88,529 | 249,726 | 226,803 | 192,582 | 138,802 | 104,275 | 85,250 |  |
|  | IS19-20 | - | Field blank | 161,730 | 135,388 | 7,172 | 59,899 | 53,177 | 22,122 | 149,736 | 102,926 | 4,446 |  |

n.a. not analysed
