# Supplementary material for: Seasonality of Glacial Snow and Ice Microbial Communities
Source: Front Microbiol. 2022 May 16;13:876848. doi: 10.3389/fmicb.2022.876848 (PMC9149292; doi:10.3389/fmicb.2022.876848)
Supplement: Supplementary file 3 [file Data_Sheet_1.docx]

Supplementary Material

**Supplementary Table S1|** Sequencing statistics of analyzed genes showing raw, quality filtered and denoised/chimera checked/contamination filtered sequences.

**Supplementary Table S2.** Marker gene (16S and 18S) sequencing counts for all sample sites including taxonomic assignment and reference sequences. This table is provided as a Microsoft Excel file (Table S2).


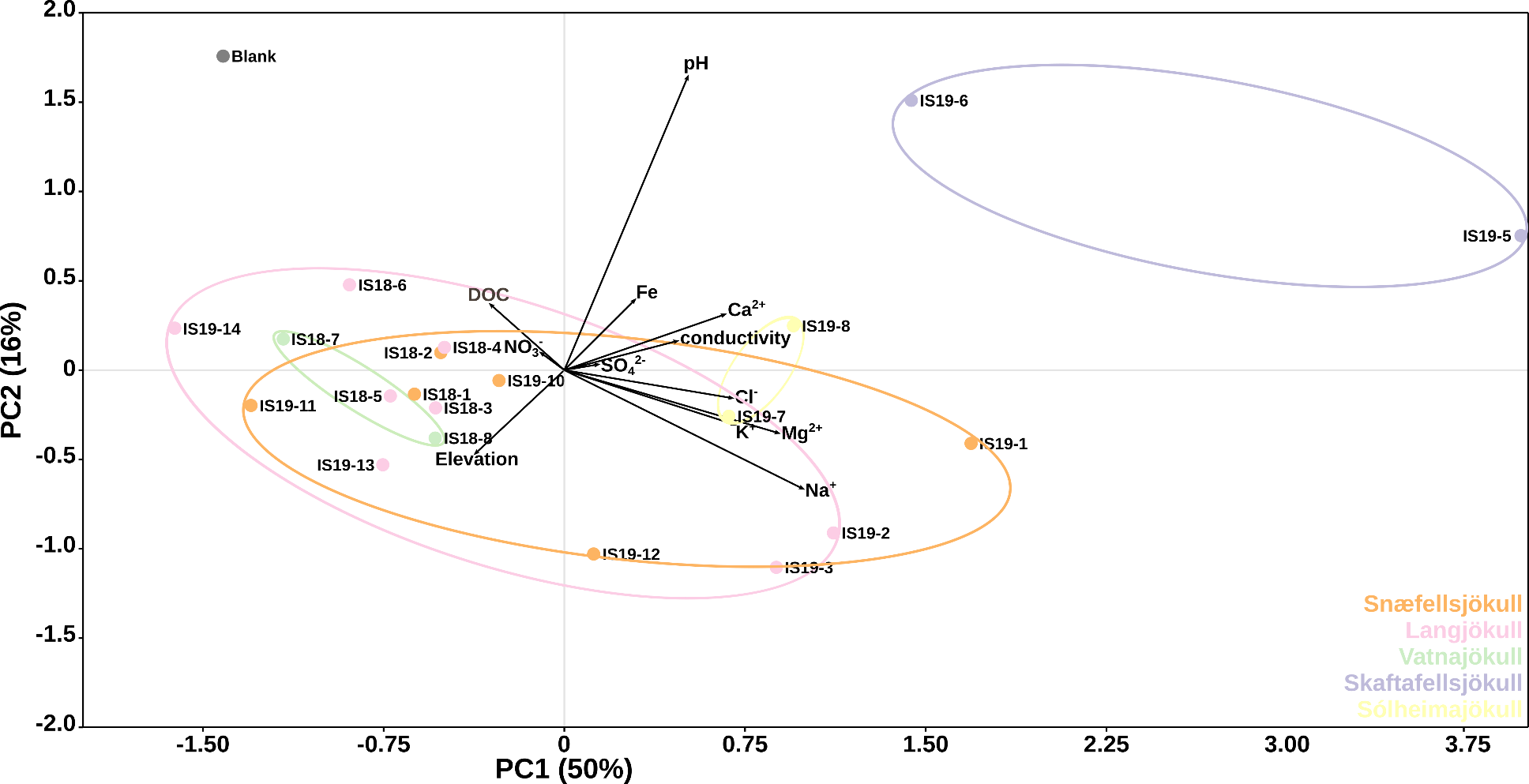


**Supplementary Figure S1.** Principal component analysis of environmental and geochemical parameters by location. Black vectors show different environmental/geochemical parameters whilst differently colored dots represent location sample collection: orange for Vatnajökull, yellow for Snæfellsjökull, light brown for Skaftafellsjökull, brown for Solheimajökull and red for Langjökull. A distilled water field blank is shown in gray. The first and second axis explaining 50% and 16% of the variation, respectively. Ellipses have been drawn for guidance.


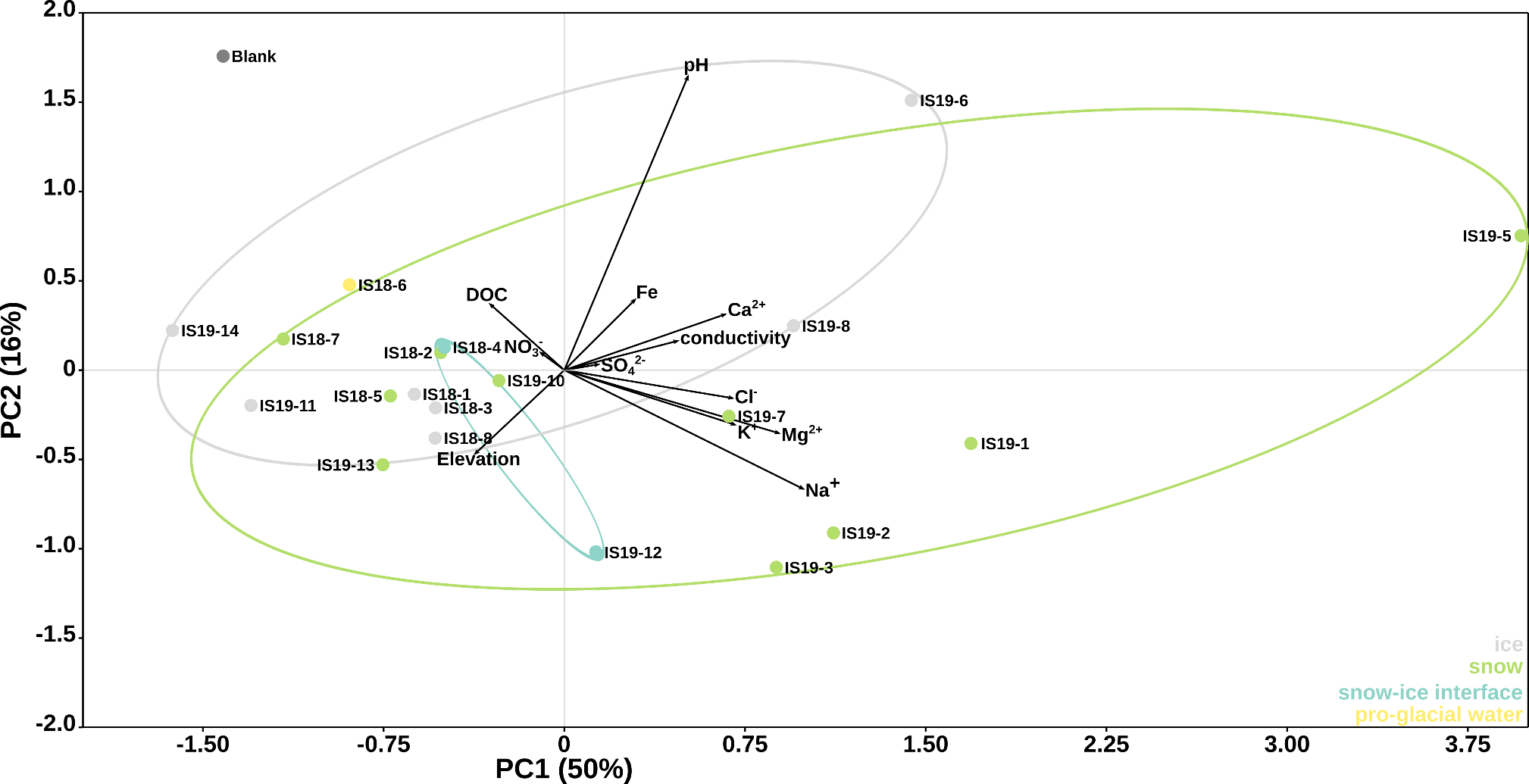


**Supplementary Figure S2.** Principal component analysis of environmental and geochemical parameters by habitat. Black vectors show different environmental/geochemical parameters whilst differently colored dots represent sample types: blue for snow-ice interface , light blue for snow, dark blue for ice and sky blue for pro-glacial water. A distilled water field blank is shown in gray. The first and second axis explaining 50% and 16% of the variation, respectively. Ellipses have been drawn for guidance.


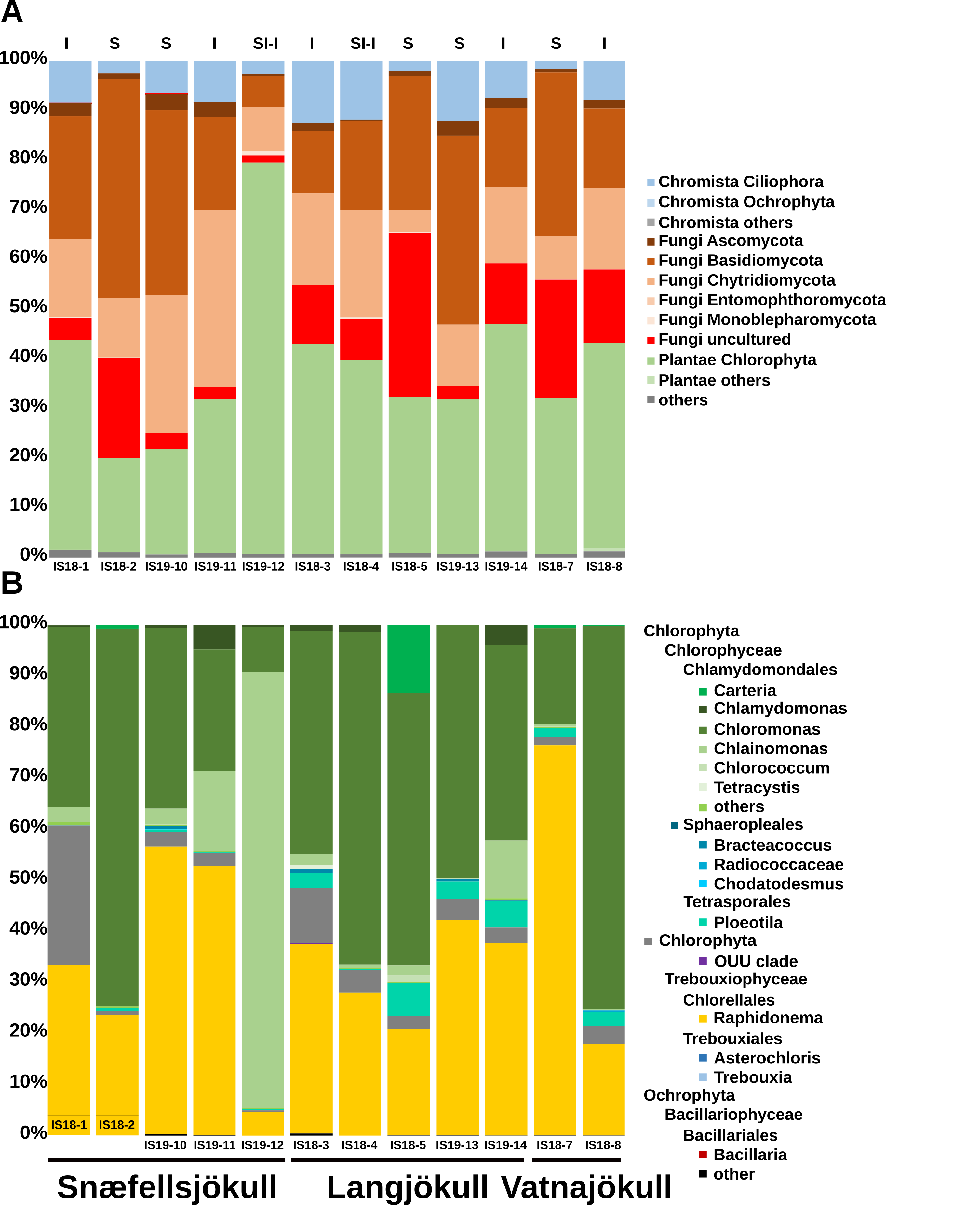


**Supplementary Figure S3.** Stacked bar-plots showing ITS2 gene relative abundance at phylum (A) and Chlorophyta at genus level (B). Samples are ordered by glaciers and habitats are indicated by letters with I for Ice, S for Snow, and SI-I for Snow-Ice interface. Only summer samples are plotted because ITS2 amplification for winter samples did not yield a product.


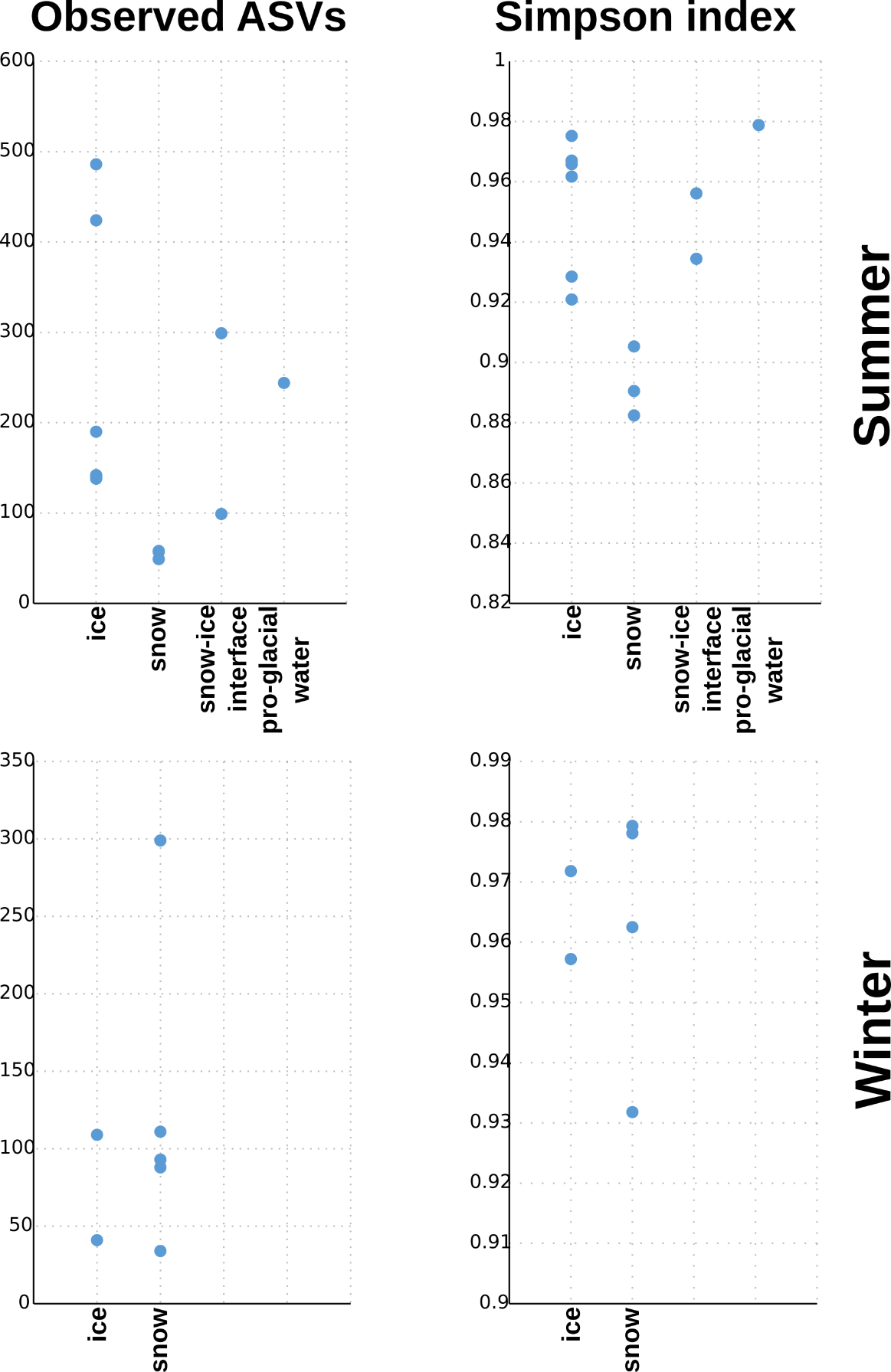


**Supplementary Figure S4.** Alpha diversity indexes based on 16S rRNA gene sequences. The upper row shows summer samples while the lower row shows winter samples. The samples have been divided into habitats ice, snow, snow-ice interface, and pro-glacial water.


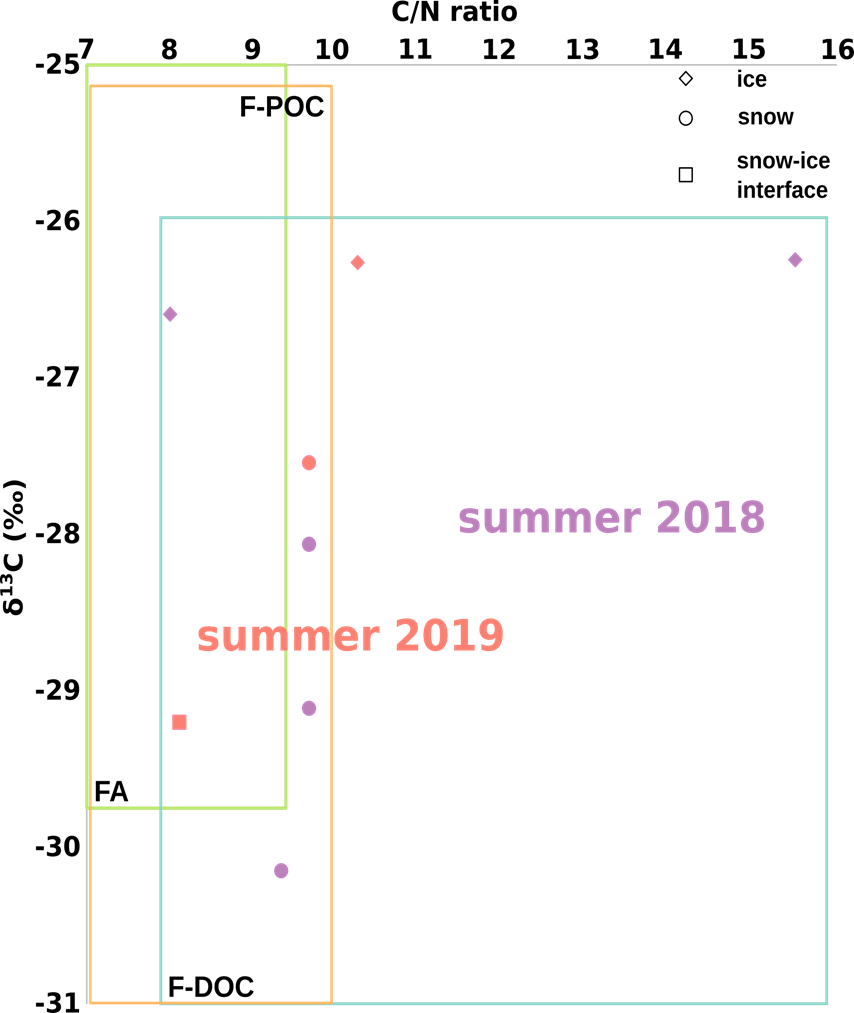


**Supplementary Figure S5.** δ^13^C and C/N ratio of snow and ice samples. Symbols represent the habitats and symbol colors represent samples collected in summer 2018 (purple) and summer 2019 (red). Colored rectangles show literature values for freshwater particulate organic matter (F-POC, orange), freshwater dissolved organic carbon (F-DOC, turquoise), and freshwater algae (FA, green), (Lamb et al., 2006).

**Supplementary Figure S6.** Heat-map showing the abundance of *Bacteriodetes* ASVs in the summer and winter samples. ASVs taxonomy is given as highest description between order to genus level. This is provided as a separate PNG image file (Figure S6).
