# Supplementary figures and images for: Seasonality of Glacial Snow and Ice Microbial Communities
Source: Front Microbiol. 2022 May 16;13:876848. doi: 10.3389/fmicb.2022.876848 (PMC9149292; doi:10.3389/fmicb.2022.876848)

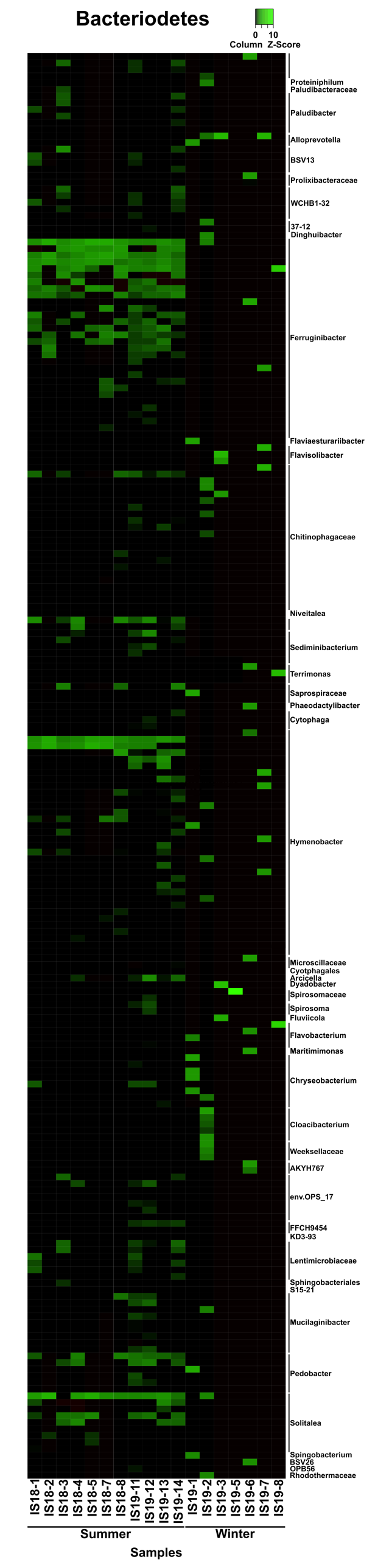

Supplement: Supplementary file 4 [file Image_6.png]
